# Effect of Myofascial Release (MFR) on Tendo Achilles (TA) Flexibility in Nurses: A Review

**DOI:** 10.7759/cureus.31319

**Published:** 2022-11-10

**Authors:** Ishika U Chauhan, Priyanka A Telang

**Affiliations:** 1 Community Health Physiotherapy, Ravi Nair Physiotherapy College, Wardha, IND

**Keywords:** ta tightness, self-myofascial release, ankle injuries, tendo achilles, myofascial release technique

## Abstract

Increased muscle stiffness can result in a reduced range of motion (ROM) and impaired function. An increased ankle injury risk has been associated with reduced ankle dorsiflexion ROM. Although self-myofascial release (SMFR) is commonly used in clinical and sporting settings, little is known about its impact on gastrocnemius and Achilles tendon (AT) stiffness. As a result, we investigated how SMFR using a foam roller (FR) affects gastrocnemius-AT complex stiffness and ankle dorsiflexion ROM. According to these results, self-myofascial relaxation of the gastrocnemius and an improvement in ankle dorsiflexion ROM can be accomplished by rolling an FR along the calf. Myofascial release (MFR) is a type of manual therapy that involves stretching the myofascial complex with a light load and for a long duration in order to restore optimal length, relieve pain, and improve function. Viscoelastic deformation is the rapid increase in muscle length following stretching.

## Introduction and background

**Figure 1:**
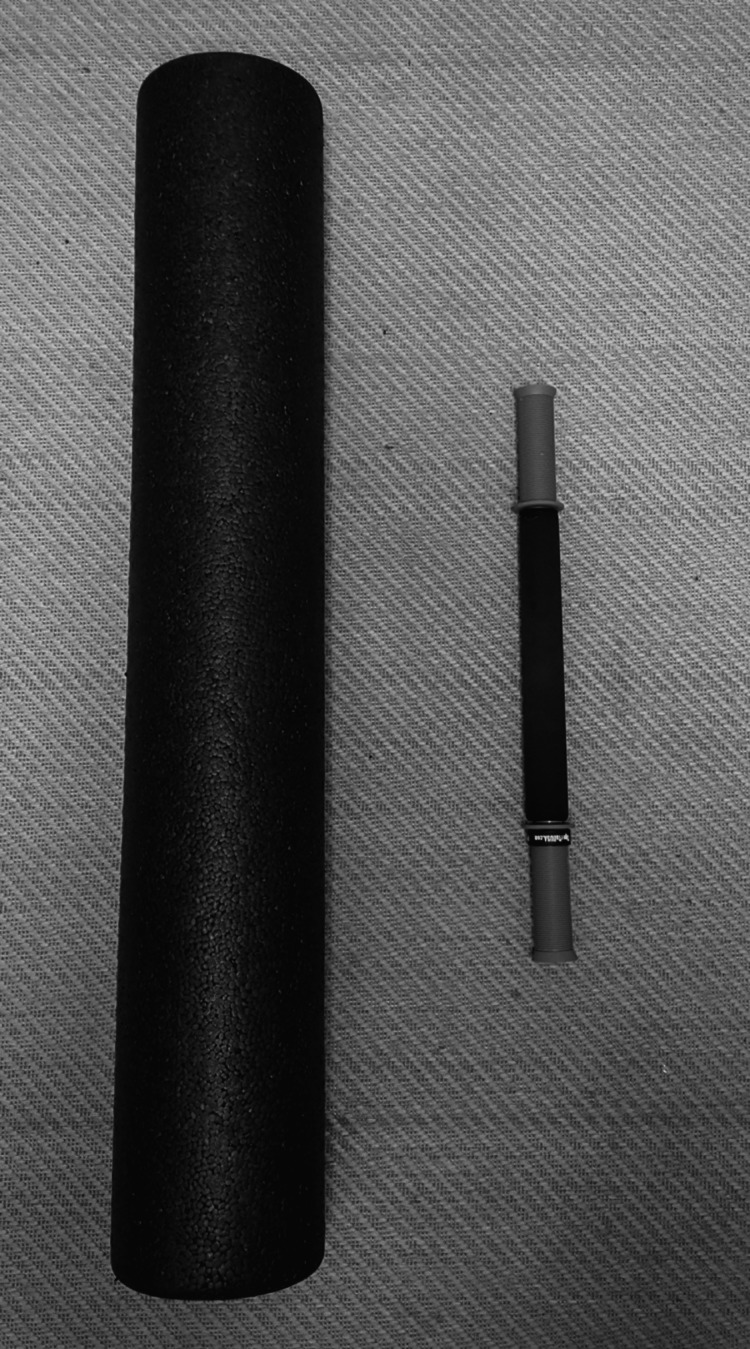
Self MFR tools. MFR, myofascial release

As a result of their heavy-duty, nurses are subjected to significant physical and mental stress, and they are not even covered by health insurance. The majority of nurses are obliged to work long shifts. Workplace standing for extended periods has been linked to several potentially serious health effects, including lower back and leg pain, cardiovascular issues, weariness, discomfort, and pregnancy-related health issues. Long periods of standing at work have been related to a variety of serious health problems [1]. Due to serious and long-standing challenges, nurses at Tehran's burn centers have been burned out. This incident provoked significant reactions and put nurses, patients, and the organization at risk. Managers and nurse executives have a hard time figuring out what factors influence nurses' burnout responses and designing delivery strategies that encourage positive adaptation and support improved quality care. This study, which is part of a larger study, attempts to investigate and describe nurses' perspectives on the factors influencing their burnout responses [2]. The powerful effect of good personal attributes, as well as its sensitivity to long-term and intense organizational demands, proposes strategies for implementing stress reduction programs and revitalizing nurse morale by emphasizing ethical components of caring [3]. Patients can use the rollers to isolate certain portions of the body and alleviate limitations in the soft tissue by changing their body positions. Foam rolling before an exercise, like massage, is supposed to assist in re-establishing muscle length tension connections [4]. In addition to enhancing flexibility and reducing perceived muscular pain, massage and stretching decrease motor unit activity. During strenuous physical exercise, fibrous adhesions are thought to develop as a result of trauma at a small level. It has been demonstrated that these adhesions interfere with the normal function of the muscle, including a joint range of motion (ROM), muscle length, muscle coordination, and diminished strength and power generation. Treatment for fascial fibrous adhesions using massage and comparable methods is thought to be beneficial [5]. Myofascial release (MFR), a type of massage, is a recent method to improve athletic performance. To lessen fibrous adhesions that develop between fascial/connective tissue layers, Barnes developed this approach. According to theory, these fibrous adhesions are caused by microtrauma, inflammation, excessive muscle activation, muscular imbalances, and trauma. Myofascial trigger points, often known as "tight places in the muscles," are described as "hypertensive palpable nodules or taut bands" of muscle tissue that are frequently found in the muscle belly. Massage and MFR treatments are used to dissolve these fibrous adhesions.; however, the drawback of these methods is that they are typically quite time-consuming [6]. Self-myofascial release (SMFR), a method that resembles therapeutic MFR, is believed to offer benefits comparable to those of the latter. SMR practitioners, on the other hand, differ in how they apply pressure to the desired area by either using their body weight or leverage. Using foam cylinders, foam rolling has been recently incorporated into training routines (foam rollers, FR). On applying pressure to the desired area, the user just places their back, buttocks, or thigh on one of the FRs while rocking back and forth FRs and roller massage bars come in several sizes and foam densities (Figure 1) [7].

As previously stated, tendo Achilles (TA) tightness can hurt lower limb function, resulting in abnormal foot arches and a higher risk of falling. Tightness of the TA and improper bone alignment pathological alterations in the foot and ankle are caused by the major deforming force of the foot and ankle structures [8]. MFR is a type of manual therapy that involves restoring the myofascial complex to its ideal length, reducing pain, and enhancing function by stretching it for a prolonged period while applying a light load [9]. MFR has been used by therapists and fitness professionals as a rehabilitation or maintenance aid over the past decade, primarily through foam rolling. The MFR approach resulted in a considerable increase in joint ROM, but no loss in muscle force changes in performance, according to the researchers. For a long time, MFR treatment has been utilized to treat myofascial pain. The impact of MFR on myofascial pain, however, has not been the subject of any clinical studies [6]. The ankle and foot complex is essential for maintaining an erect posture as well as for acclimatizing to supportive surfaces, correcting postural sway in a single-limb stance, absorbing stress, and ground reaction force (GRF) transition during regular locomotion [10]. Flexibility is regarded as an important component of normal biomechanical performance in sports. Lack of flexibility can cause early muscle exhaustion or change typical movement biomechanics, putting you at risk for injury [11]. Flexibility is defined as the total ROM in a joint(s) as well as the length of muscles that span the joint. Pain syndrome and balance difficulties might be exacerbated by a loss of flexibility. Flexibility is influenced by several factors: training oversight, muscular strength, endurance, and ROM, as well as genetics, are all internal elements. Weather, age, walking surface, and shoes [12]. An increase in Achilles tendon (AT) tension is accompanied by an increase in plantar fascia strain. Overstretching of the AT as a result of strong muscle contraction and passive stretching of a tight AT are both possible mechanical causes of plantar fascia overstrain [13]. Foam rolling, also known as MFR, is a popular warm-up technique in sports. Employees in the nursing field had a higher rate of lower back problems than those in other professions [14]. The foam roll and many kinds of roller massagers are typical SMFR implements. There is evidence to support the idea that these instruments can improve joint ROM and the healing process by reducing the negative impacts of acute muscle soreness, delayed onset muscular soreness (DOMS), and post-exercise muscle function. The sizes and foam densities of FRs and roller massage bars vary. Standard (6 inches 36 inches) and half-size (6 inches 18 inches) commercial foam rolls are the two sizes that are frequently offered. When foam rolling, the client applies pressure to the soft tissues by rolling while using their body weight as leverage. Roller massage bars also come in many shapes, materials, and sizes. One of the most common is a roller massage bar constructed of a solid plastic cylinder with a dense foam outer covering [15]. This study's goal is to analyze the most recent research on AT injuries, specifically chronic tendinopathy and acute ruptures, in terms of their causes, diagnoses, available treatments, and results [16]. FRs and roller massage bars come in several sizes and foam densities (Figure 1).

## Review

Methodology

Database searches were done for relevant peer-reviewed articles, using the keywords TA tightness, MFR, ankle injuries, and TA. Articles were screened and relevant articles were included in the study. Research papers, original articles, systemic reviews, literature reviews, case-control studies, randomized trials, and cross-sectional studies were considered. FRs are used in MFR, which includes a wide variety of therapy techniques, including massage and self-massage. The review article carries out a study on the influence of MFR on TA flexibility by evaluating and categorizing them with the help of FPI (foot posture index) based on inclusion and exclusion criteria. FPI was evaluated initially and was graded (-2) for which stretching is given followed by the hot pack for five days and then a gap of one day is given for evaluating the muscle; no changes were seen which is why the same session was continued for 4 weeks; then after 4 weeks the muscle was evaluated again and it was graded (+2) and the changes were seen in the muscle; further ergonomics advice was given.

Incidence

The incidence of AT rupture in the general population is approximately 5-10 per 100,00. AT injuries are becoming more common, although surgical intervention is becoming less necessary due to advancements in non-surgical therapies, which may provide comparable results without the associated surgical risk. Open and minimally invasive procedures are the same when surgery is involved. Most patients can return to their pre-injury level of activity, with the unfortunate exception of the elite athlete [17].

Predisposing factors

Poor body mechanics, training mistakes, environmental conditions, or systemic diseases like gout and ankylosing spondylitis can all lead to AT overuse injuries. They can also be brought on by environmental causes. Training errors were revealed to be the root cause of hyperpronation in 56% of runners, inadequate gastrocnemius-soleus flexibility in 75% of runners, Achilles tendinitis in 75% of runners, poor gastrocnemius-soleus flexibility in 39% of runners, and unsuitable footwear in 10% of the runners [18].

Clinical features

A thorough history and clinical examination, which includes watching the patient walk and looking at their shoes, should be the foundation for a reliable diagnosis of Achilles tendinitis. When a patient reports an AT injury, a thorough history must be taken. Changes in physical activity level and footwear, in particular, should be sought after. It is important to determine the severity of the patient's symptoms. In the more persistent cases, morning stiffness has been reported. Pain during acceleration or sprinting nearly always corresponds with tendinosis or a partial rupture found during surgery [19]. The level of tendon involvement influences the physical findings. Common results include tight hamstrings and decreased ankle dorsiflexion. The inferior portion of the posterior calf is where the pain is concentrated. Usually, 3-6 cm above the tendon insertion, there is tenderness on palpation. Tendonitis is thought to manifest as a sensitive, nodular swelling, which is typically prevalent in people who have more persistent symptoms [20]. It could be necessary to use an orthotic to treat any underlying heel valgus and assist the arch return to normal. With a 3/8-inch heel wedge, patients can go about their daily lives with fewer symptoms. Before a patient with paratendinitis, tendinosis, or a partial rupture can fully return to sports, he/she may need to get conservative care and undergo rehabilitation for several months [21].

Treatment

Myofascial release employs manual manipulation to ease pain and promote healing. Fascia can become restricted as a result of trauma, stress, injuries, and poor posture. Release of fascia constriction and tissue restoration are the two main objectives of MFR. By employing this method, the fibrous fascial bands of connective tissue can be relieved of pressure. It is thought to dissolve adhesions, soften, and extend the fascia by gently and continuously stretching it [22]. MFR, which releases fascia that may be impending blood arteries or nerves, is also thought to boost the body's natural healing abilities by enhancing circulation and nervous system communication. Some practitioners claim that the technique also releases suppressed emotions (since emotions are stored in the fascia, often releasing the fascia physically can bring up emotions stored in that area from the original trauma) that may be causing the body to experience tension and suffering. The fascia softens, lengthens, and realigns as a result of movement, which has been compared to kneading a piece of taffy. On the constricted fascia, the direct MFR approach is effective. By stretching, lengthening, or mobilizing sticky tissues, MFR attempts to alter the myofascial structures [23].

Etiology

Tendon Achilles overuse injuries are well documented and very prevalent. Repetitive overloading of the AT above its physiological threshold can result in sheath inflammation, body degeneration, or a combination of the two. Currently, it is uncertain what causes Achilles tendinopathy. Several factors, including usage, poor vascularity, lack of flexibility, genetic makeup, gender, endocrine or metabolic abnormalities, and quinolone antibiotics, have been related to tendinopathies. The main pathogenic cause is currently believed to be excessive tendon loading during physical exercise [24]. If a tendon is repeatedly overloaded above its natural limit, it will respond by inflaming its sheath, degrading its body, or a combination of the two. Even if within physiological limitations, repetitive micro trauma to the tendon without appropriate time for recovery and healing might result in tendinopathy. Microtrauma is related to uneven tensions between the gastrocnemius and soleus because of their distinctive unique contributions to force [25]. This results in asymmetric stress distributions inside the tendon, as well as frictional forces between the fibrils and localized fiber damage. It will almost certainly be complicated, with both intrinsic and external factors at play. Sports injuries can be caused by intrinsic or extrinsic causes acting alone or in combination. Vascularity, gastrocnemius soleus complex dysfunction, age, gender, body weight and height, pes cavus deformity, and lateral ankle instability are all inherent variables [26]. Extrinsic factors that cause tendinopathy include changes in training patterns, poor technique, past injuries, footwear, and environmental conditions such as training on hard, slippery, or slanting surfaces. In the case of acute damage, extrinsic causes predominate; however, overuse injuries and chronic tendon problems are frequently complex in nature Achilles tendinopathy and is caused by a variety of factors [27]. In epidemiological research, two-thirds of athletes with AT issues had diverse patterns of lower extremity malalignment and biomechanical defects. The prevalence of such issues is 60%. However, the processes through which these factors contribute to Achilles tendinopathy development are unknown. Hyper pronation of the foot is the most prevalent ankle misalignment [28]. Athletes with Achilles tendinopathy had lower subtalar joint mobility and lower ankle joint ROM than athletes with other symptoms. Achilles tendinopathy is also connected to forefoot varus, higher hindfoot inversion, and decreased ankle dorsiflexion with the knee in flexion. Excessive frontal hindfoot movement, especially a lateral heel strike with excessive compensatory pronation, is hypothesized to provide the AT a "whipping action," putting it at risk for tendinopathy [29].

Pathogenesis

The pathophysiology of AT problems is complicated by a number of factors, including tissue hypoxia, free radical changes caused by ischemia-reperfusion damage, and exercise-induced hyperthermia. When a tendon is stretched above 4% of its initial length, it loses its elasticity and becomes more vulnerable to a collagen structural rupture [30]. Type III collagen mRNA levels in the tendinopathic AT can be significantly higher when compared to normal samples. The significance of this discovery is currently being debated. It is important to note that the majority of the following elements should be regarded associative rather than causative evidence, therefore, their role in the disease's development is still up for debate [31]. We have seen a lot of AT patients with various sorts of illnesses. Some ruptures occur without any warning signs or symptoms. Others describe weeks or months of pain and functional limitations before the rupture. Others show signs of peritendinitis, which is the most common cause of sports-related impairment [32]. Many types of AT illnesses that do not respond to conservative treatment are treated surgically because of the pain and dysfunction they cause. As a result, we were able to investigate the histology of these various clinical complexes. We observed just a thickening of the peritendon that was adhering to normal tendon tissue when we looked at peritendinitis macroscopically [33]. The histologic examination revealed capillary proliferation in the peritendinous tissue, as well as the formation of inflammatory cells, indicating that the tendon and peritendinous tissue are adherents. The tendon itself was entirely normal in all of these situations. The macroscopic findings in other cases of peritendinitis revealed the existence of degenerative processes (tendinosis). The tendon was thicker, softer, and yellowish, and it had lost its lustre. Fibrillations and tiny areas of cleavage were identified in various portions of the tendon. Histologically, not only was peritendinous tissue inflammation visible but also there were also distinct areas of localized tendon degradation. The presence of a significant vascular proliferation on the peritendon, which tended to invade the normal tendon, was of particular interest.

## Conclusions

Our results suggest that a single calf FR intervention can improve ROM of ankle dorsiflexion and decrease stiffness in the gastrocnemius. In healthy individuals, the current study discovered a negative relationship between ankle dorsiflexion ROM and AT stiffness. Stretching allegedly improves flexibility by reducing reflex activation and raising stretch tolerance. Stretching has an immediate impact due to viscoelastic deformation.
